# Results of Strict Sanitary Regulations in Arizona1Read before the American Veterinary Medical Association, Minneapolis, Minn., September, 1902.

**Published:** 1902-10

**Authors:** J. C. Norton

**Affiliations:** Territorial Veterinarian, Phœnix, Arizona


### RESULTS OF STRICT SANITARY REGULATIONS IN ARIZONA.[1]

By J. C. Norton, V.S.,

TERRITORIAL VETERINARIAN, PHŒNIX, ARIZONA.

When first asked to write a paper on this subject to be read before this Association of veterinarians, who are more largely interested in the practice of veterinary medicine than in sanitary work, I thought it could only be considered that it was another Westerner trying to praise not only the Territory in which he lives, but even the work that he was largely responsible for in that Territory, for it happens that I have been closely connected with the sanitary work of Arizona during the past nine years.

However, as all of us learn and are encouraged largely from the experiences of others, it may be that some of you may receive a little benefit in the next few minutes from the experiences of a Western Territory. As we are located in the extreme Southwest, "Woolly West" you Easterners call it, it will not be expected that I will give anything new, and, in fact, the topic does not indicate that I will give anything other than results.

Owing to the cautious action of the members of the United States Congress, due mainly, I think, to their lack of knowledge of exact conditions existing, Arizona is one of three Territories still to be admitted to the Union. Even though Arizona is a Territory her people have been awake to their best interests, and some fifteen years ago passed live-stock sanitary laws, which have been improved from time to time, until now the regulations governing the suppression of contagious diseases in the Territory and the admission of live-stock into her limits are as strict as those of any State or Territory in the Union.

Arizona's present sanitary laws require the Governor of the Territory to appoint a Sanitary Board composed of three stockmen, who receive per diem and mileage when on duty, a secretary for said Board, who receives a salary, and a Territorial Veterinarian, who must be a graduate of a recognized veterinary college, who also receives a salary and mileage. These officers are given power to make and enforce any regulations they deem necessary for the prevention, control, and suppression of contagious diseases among the live-stock of the Territory. No certain

[1] Read before the American Veterinary Medical Association, Minneapolis, Minn., September, 1902.

amount is appropriated for their work, but their accounts are paid from the general fund of the Territory.

Though Arizona is a mountainous country and has extensive mining interests, yet hundreds of thousands of well-bred cattle, sheep, and horses are shipped annually from her ranges. Many of the irrigated valleys are largely devoted to the breeding of thoroughbred horses, improved breeds of cattle and swine, and the fattening of range-bred cattle for Eastern markets.

Arizona touches old Mexico along its entire southern line, California along most of its western boundary, and adjoins New Mexico on the east. The Territory is quite mountainous, yet a large part of the southern portion is of low altitude and has a semi-tropical climate. With the United States quarantine line separating Arizona from old Mexico—a country with few or no sanitary laws—on the south, and from quarantined Southern California on the west, it can easily be seen that strict sanitary regulations only have kept the Territory above the Federal quarantine line.

When rigid sanitary work was commenced in Arizona, some ten years ago, there was a small amount of Southern cattle fever infection in one of the irrigated valleys that had been introduced by shipments of breeding stock before United States quarantine regulations were as strict as now; glanders was quite prevalent among horses in certain valleys, and scabies was quite generally distributed among her thousands of sheep as it was at that time in other range countries. Thus it will be seen that even in this new country contagious diseases gained a foothold early in its existence.

A rigid quarantine was placed on the districts where glanders was found, and by careful examinations, aided by the reliable mallein test, in a single year forty-nine glandered horses were found and destroyed. The stockmen of the communities gave every assistance, and the work was done so systematically that not a single case has since been detected in these districts. There have been but three mild outbreaks of glanders in the Territory since 1894, and in these cases the infection was traced directly to the horses that had been driven overland into the Territory. If our range horses had become infected with glanders, it would have been extremely difficult to stamp out the disease, not only because range horses are hard to control, but for the additional reason that in a mild climate the disease usually assumes a chronic form and is often hard to detect until an animal has possibly infected several others.

The tick (Boophilus annulatus) has reached such a high state of development—*i. e.*, in the opinion of Government and State sanitary officials—that the presence of a few will prevent large numbers of the bovine species from enjoying the green pastures and well-filled corn-cribs of the Northern States. I am not opposing the position the fever tick has been given, for its bad qualities have been proven and it is rightly placed, and for a Southern State to be free of ticks is certainly gratifying.

The Southern fever infection was found in only one irrigated valley, where the lands were under fence. There was but a small amount of infection, and yet it could only be eradicated through sanitary regulations, as climatic conditions favored its continuance. In this particular valley the extreme dry heat of summer was more detrimental to the propagation of the tick than the mild winters. Experiments proved that irrigation was not as detrimental to the propagation of the tick as would be supposed. The tick infection on these fields was eradicated by moving all cattle and horses from the same and holding the lands in strict quarantine until all infection was destroyed, which required from ten months to two years' time, according to local conditions.

Comparatively little systematic attention was given toward freeing the Territory from the pest of sheep husbandry—the scab mite—until within the last few years, when the Department of Agriculture has required that all sheep offered for interstate shipment should pass rigid inspection. Though progressive sheepmen have done effective work, this forced upon all breeders of the Territory the importance of sanitary work, and they were more willing to listen to the argument that scabies was due not to filth and poor feed, but to an animal parasite. During the last two years the amount of scabies infection has been reduced to a minimum, but under the methods by which sheep must be handled in range countries—that is, driven from place to place on account of feed and water—it is not possible to make as complete a success of the work in a given amount of time as where stock are confined in one locality.

I am glad to report that we have not had to contend with hog cholera except to prevent its introduction, as many sanitary officials have in the Middle and Eastern States, but the swine-breeders' interests suffered largely from two bad outbreaks of swine plague. Swine are raised principally on alfalfa grass in the warmer climates at comparatively little expense, and are, as a rule, free from disease. Because of these conditions breeders are not usually prepared for the small amount of stormy weather which we have each winter. Swine plague usually appears among pigs and young hogs that have been exposed in stormy weather without proper shelter and feed. Unless checked at once the disease will generally gain in virulence and spread until it destroys swine of all ages. Without assistance usually less than 10 per cent. of a herd will survive the disease and become immune. Usually the disease is stamped out by destroying all diseased animals, burning the carcasses of same, and removing healthy stock to non-infected fields and giving good food and shelter.

I think, isolated as Arizona is, we are in position to have positive proof that the theory advanced years ago by the Bureau of Animal Industry is entirely correct, that there are two distinct contagious diseases among hogs—the one, hog cholera, affecting principally the intestines, and the other, which is termed swine plague, affecting primarily the lungs. I have held hundreds of post-mortem examinations on diseased hogs in the Territory, and have failed to find a single instance where there was any indication of the ulcers on the intestines and other lesions most prominent in hog cholera, yet in all the lungs were more or less affected. Though many lines of treatment have been used by swine-breeders, especially of the Salt River Valley, yet they have proven of little value to the pigs that were affected, but tonic treatment has been of some assistance in warding off the disease when given to healthy hogs. The Government prescription has been used with best results. The sanitary precautions were of the greatest benefit, and where swine-breeders could be persuaded, when the disease first appeared in their herds, to destroy and burn the carcass of every pig that was affected and remove all that were in apparently perfect health to new fields, and those that were not in the best of condition to other new fields, giving them some extra care, the disease could usually be stamped out at once with a loss often of less than 5 per cent.

Though buzzards are protected by law in many States because of their scavenger qualities, yet they caused serious trouble in Arizona where swine plague was prevalent by carrying bits of infection from the carcasses in infected fields to healthy herds. This could only be avoided by strict police regulations requiring every stockman to promptly and thoroughly burn the carcasses of all animals dead or killed on his ranch.

Though the above history shows that strict sanitary work has been of assistance to the stock interests of Arizona, it is no more than has been done in many another State; but the part of the sanitary work in Arizona that I think has been by far the most beneficial is the protective regulations or those guarding against the admission of infected animals from other States or countries. Arizona's sanitary conditions are as favorable to the health of domestic animals as people, for which it is noted, but we are not inviting the introduction of infected animals with a view to their recovery, though we secure breeding and dairy stock from all parts of the Union.

Strict rules have been enforced during the past eight years requiring that live-stock of all classes, shipped or driven into or through the Territory, must receive the written permission of the Territorial Veterinarian before entering the Territory. These rules and regulations require all railroad and express companies before shipping any live-stock into the Territory to report the shipment to the Territorial Veterinarian, stating the point of origin and destination of same, and whether or not accompanied by a certificate of health, and if so by whom signed. Very few shipments are allowed to enter the Territory except those accompanied by satisfactory certificates of health, which are usually given by Government, State, or County veterinarians at point of origin, or that pass the inspection of the Territorial Veterinarian. Arizona not only has climatic conditions favorable to the propagating of many infectious diseases—anthrax, black-leg, Southern fever, etc.—when one introduced, but is particularly liable to receive infection because a large portion of her dairy and breeding stock is shipped from Southern States. Besides hundreds of shipments of cattle pass through the Territory by rail, unloading en route, some originating below the quarantine line in Texas and California destined for points below the quarantine line, and many others originating north of the quarantine line pass through portions of the infected districts of other States before reaching Arizona, making it very important for her interests as well as the stock in transit that all these shipments be unloaded in proper corrals and handled with care.

As proof of the value and necessity for the careful enforcement of strict regulations governing the admission of live-stock into Arizona, I will state briefly conditions of three shipments of infected cattle that were shipped into the Territory during the past eight years. Only these three times during these years has infection been introduced into the Territory by live-stock that entered by rail.

In one of these cases (1894) the shipment was accompanied by a certificate of health, which was either given by an incompetent veterinarian or without due investigation, for fully 60 per cent. of the shipment, which was dairy stock, proved to be tuberculous. Fortunately the cattle were examined within two weeks after arrival and placed in quarantine until all infected animals were destroyed. Certainly no veterinarian should give a certificate of health without positive personal proof that animals in question are healthy.

During the same year a shipment of Southern cattle was brought into the Territory by rail without being reported to the proper authorities. The stock happened to be placed in an inclosed field with susceptible cattle, which in due time contracted the fever. The owner of the fields reported this fact to the Territorial Veterinarian, claiming that the cattle were dying from the effects of bad water. The cattle and fields were placed in quarantine until all infection was eradicated, which would have been practically impossible if the cattle had been placed on the open range.

The third instance occured in 1897, when a train-load of infected Southern cattle was shipped to a town just inside the eastern border of Arizona during the open season (December 31), the railroad officials claiming that the Federal government allowed Southern cattle shipped during that period without restrictions. The cattle were placed in quarantine in the railroad corrals as soon as unloaded, with orders that they should not be removed until free from infection. Though there was snow on the ground when the cattle were unloaded and stockmen of the community objected to having them held in quarantine, it proved to have been a wise precaution, for they were not entirely free from fever ticks until after the seventy-ninth day. At this time the weather conditions were favorable to the propagation of the tick, and the stockmen of that locality paid the owner liberally to ship the cattle out again rather than to have them placed on their range even free from ticks.

This remarkably slow development of the tick was largely due to the cattle being in poor condition and receiving very little care while in quarantine. They had not become reinfected, for the corrals were thoroughly disinfected every fifteen days during the time the cattle were in quarantine. Incidentally I will state that Arizona's rules have forbidden the admission of tick-infested cattle during all seasons for the past seven years, and Southern cattle entering during the open season must pass inspection and be held in quarantine in fenced fields at destination for at least twenty days and then pass a second inspection. This quarantine rule insures our ranges against fever infection from other States.

The regulations governing the admission of live-stock into Arizona are enforced without detaining shipments and at no expense to the shipper when proper certificates are presented. Our regulations are clear, and as they are published by all railroads west of the Mississippi River, few shipments are now offered that are not accompanied by proper health certificates.

Because of range conditions in Arizona the stock laws require that all cattle, horses, sheep, and swine shipped out of the Territory or slaughtered within the Territory be inspected for marks and brands by an inspector appointed by the Sanitary Board, and the shipper must show proof of ownership. This gives an additional opportunity to trace up any infection existing and to guard against infected stock being shipped out of the Territory. The Territorial Veterinarian and Sanitary Board of Arizona are responsible for the movements of all cattle into and out of the Territory, and under the above-described methods they can be thoroughly familiar with the history of the stock in any community, and, therefore, are in better condition to locate infection.

According to Arizona laws all live-stock sanitary regulations are enforced by the Territorial Veterinarian, under the direction of the Live-stock Sanitary Board, and all infection, whether detected by veterinarians or laymen, must be reported to those officers. I have observed that in some States conflicting city, county, and State regulations have prevented the best results. I am pleased to state that the few veterinarians in Arizona have given their assistance to sanitary work.

As a result of the efforts of the sanitary authorities of Arizona, I can state that no cases of anthrax or tuberculosis have been found in the Territory during the past two years, but two glandered horses have been destroyed and none are in quarantine, and the valleys that for years had some fever infection are now freed and no swine or sheep are in quarantine at the present time. I do not wish anyone to suppose that I am claiming that similar rules would be necessary or practical in other States, but have simply given methods used and results obtained in Arizona.

---

Drs. W. C. Fair, of Cleveland, Ohio, and W. Horace Hoskins, of Philadelphia, Pa., were both seen in the Buffalo Horse Markets in September.

